# A prospective observational study of urinary cytokines and inflammatory response in patients with Overactive Bladder Syndrome

**DOI:** 10.1186/s12894-021-00809-4

**Published:** 2021-03-19

**Authors:** Kiren Gill, Harry Horsley, Sheela Swamy, Rajvinder Khasriya, James Malone-Lee

**Affiliations:** 1 Women’s Health, Whittington Health NHS Trust, Magdala Avenue, London, N19 5FN UK; 2 Bladder Infection and Immunity Group (BIIG), Department of Renal Medicine, Division of Medicine, University College London, Royal Free Hospital Campus, Rowland Hill Street, London, NW3 2PF UK; 3 Women’s Health, St Thomas’ Hospital, Westminster Bridge Road, London, SE1 7EH UK; 4 Bladder Infection and Immunity Group (BIIG), Department of Renal Medicine, Division of Medicine, University College London, 10 Harley Street, London, W1G 9PF UK

## Abstract

**Background:**

Contemporary studies have discredited the methods used to exclude urinary tract infection (UTI) when treating overactive bladder (OAB). Thus we must revisit the OAB phenotype to check that UTI has not been overlooked.

**Aims:**

To examine the differences in urinary cytokines IL6 and lactoferrin in OAB patients compared to controls, with references to microscopy of urine and enhanced quantitative urine culture.

**Methods:**

A blinded, prospective cohort study with normal controls using six repeated measures, achieved two-monthly, over 12 months.

**Results:**

The differences between patients and controls in urine IL6 (F = 49.0, *p* < .001) and lactoferrin (F = 228.5, *p* < .001) were significant and of a magnitude to have clinical implications. These differences were for lactoferrin correlated to symptoms (9.3, *p* = .003); for both to pyuria (IL6 F = 66.2, *p* < .001, Lactoferrin F = 73.9, *p* < .001); and for IL6 microbial abundance (F = 5.1, *p* = .024). The pathological markers had been missed by urinary dipsticks and routine MSU culture.

**Conclusion:**

The OAB phenotype may encompass patients with UTI that is being overlooked because of the failure of standard screening methods.

## Background

Overactive bladder syndrome (OAB) is characterized by urinary urgency, with or without urgency urinary incontinence, usually with increased daytime frequency and nocturia, if there is no proven infection or other obvious pathology [1]. The diagnosis is contingent on exclusion of urinary tract infection (UTI), usually achieved by bedside urinary dipstick testing and/or midstream culture analyses using Kass criterion for exclusion of infection [2]. Recently, dipstick and culture testing have been discredited due to poor sensitivity and ambiguous and misleading culture data [3, 4]. This raises the possibility that the OAB may need to be revaluated.

We have published data from fresh urine microscopy [5], routine culture and spun urinary sediment culture [3, 6]. We reported significant differences in the urinary white cell and urothelial cell counts and in the results of spun sediment culture between OAB patients and normal controls, over a period of 12-months. Like others [4, 7], we found that the dipsticks and routine culture failed to discriminate between the OAB patients and normal controls. However, other surrogate markers of infection and inflammation: Spun sediment cultures, microscopic pyuria (WBC), and urinary uroepithelial cell (EPC) counts, revealed significant between group differences [5]. These data suggest that standard screening for UTI in the assessment of patients with OAB may be misleading when excluding infection.

Given the implications of these findings, we set out to validate our observations by comparing those three surrogate infection markers against the behaviour of two key urinary cytokines namely IL6 and Lactoferrin, in patients with OAB. We have already published on ATP in this context [8]. We chose IL-6 because it is a well-established inflammatory marker, and it has been reported to rise in association with increased bacterial loads in UTI. Lactoferrin acts to sequester iron and thereby deprive offending microbes of vital nutrition. Increases in urinary lactoferrin have been reported in association with UTI.

## Aim

To explore the relationship between urinary cytokines and key urinary markers of bacterial cystitis; pyuria [9], and urinary spun sediment culture [10, 11] in women with OAB symptoms comparing them to normal controls, over a period of twelve months.

## Methods

We conducted a prospective, blinded, observational cohort study of female outpatients with OAB symptoms, and asymptomatic control subjects. The study groups included patients who described OAB symptoms as defined by the ICS definition [12], including urinary urgency, increased day time frequency, nocturia with or without urge incontinence. Healthy female adults matched for age and menopausal status, with no urinary symptoms were recruited as controls. All participants provided written, informed consent. Women who were pregnant or planning a pregnancy were not eligible for inclusion. We did not include patients with structural disease of the urinary tract and other systemic diseases.

Study participants attended two monthly study visits over a one year. Patients and control subjects provided midstream urine (MSU) samples using the clean-catch method. All samples were subject to analysis by blinded researchers. We assessed the patient’s symptoms, urinary cytology, microbiology and urinary immune response. Symptoms were assessed using standardised validated questionnaires including the ICIQ-FLUTS, a pain score and an urgency score [6]. Aliquots of spun urine were frozen at − 80 °C for cytokine analysis. Methods are discussed in detail below.

### Symptom collection

Symptoms were collected using validated questionnaires which were self-administered [6, 13].

### ICIQ Questionnaires

The International Consultation on Incontinence Questionnaires (ICIQ) was selected to evaluate symptoms [14].

### Urgency Score

The symptoms of urinary urgency were measured using a validated ten-item scale, recording the characteristics and the degree of urinary urgency [15, 16].

### Pain Score

The pain questionnaire, a validated, eight-item scale which recorded dysaesthesia/pain symptoms associated with the lower urinary tract [17].

## Urine analyses

The methods of the assessment of pyuria and the microbiological assessment using the spun urinary sediment culture have been reported [3, 4, 7, 9, 10]. We used unspun fresh urine microscopy to obtain pyuria. Enhanced urinary sediment cultures were performed for quantitative and qualitative microbial analysis [3]. All specimens were subject to urinary dipsticks analysis and routine NHS culture with or without sensitivity where a positive culture was declared if ≥ 10^5^ cfu/ml. of a pure culture of a known urinary pathogen.

## Urothelial cytokine response

Fresh urine samples were centrifuged and the supernatant was removed and aliquots frozen at -80 °C. For each cytokine analysis a separate aliquot was used and the urine samples only underwent one freeze / thaw cycle to ensure cytokine stability.

### Quantification of Urinary IL-6

The Quantikine® High Sensitivity ELISA Human IL-6 Immunoassay was used to quantify urinary IL-6 (R&D Systems, Abingdon, UK), with a limit of detection of 0.09 pg ml^−1^ with an inter- and intra-assay coefficient of variation of less than 10%. Frozen urine samples underwent only one freeze thaw cycle ensuring stability of IL-6. The urine samples were thawed to room temperature and mixed thoroughly using a vortex mixer (Scientific Industries, New York, USA). Urinary IL-6 was determined using an Opsys MR fluorescence microplate reader (DYNEX Technologies, Worthing, UK). The minimum detectable dose (MDD) of the ELISA kit according to the manufacturers ranges from 0.016 to 0.110 pg ml^−1^, with an average MDD of 0.039 pg ml^−1^. All samples were analysed in triplicate to test the inter-assay precision and the mean value was taken. The intra-assay precision for urine assays suggested by the company was 5.5–9.8% and the inter-assay precision for this experiment was 5.5–11.2%.

### Quantification of Urinary Lactoferrin

A separate aliquot of the frozen urine sample underwent one freeze thaw cycle to ensure stability of Lactoferrin. The Human Lactoferrin ELISA immunoassay was used to quantify urinary Lactoferrin (ICL, Portland, USA), with a range of detection of 3.125 ng ml^−1^ -100 ng ml^−1^, with a sensitivity average of 0.725 ng ml^−1^.

Lactoferrin concentration was determined using an Opsys MR fluorescence microplate reader (DYNEX Technologies, Worthing, UK). The reader produced standard curves, fit to a four-parameter logistics curve, which was used to calculate concentrates for each well. All samples were analyzed in triplicate to test the inter-assay precision and the mean value was taken.

### Primary outcome measure

The primary outcome measure was urinary IL-6.

### Secondary Outcome measures

The secondary outcome measures included the assessment of urothelial inflammation and immune activation, routine microbiological assessment and lower urinary tract symptoms. We studied the following measures:

1. Microscopic pyuria count
2. Urinary Lactoferrin
3. Qualitative and quantitative poly microbial growth using enhanced sediment cultures demonstrated on CPS3 agar
4. ICIQ-LUTS symptoms score
5. Whittington urgency score
6. Whittington pain score
7. Urine dipstick analysis
8. Routine NHS laboratory MSU culture

### Statistical analysis

The primary statistical analysis was to determine the difference in urinary IL-6 in patients compared to controls. The secondary analyses similarly compared urinary lactoferrin and explored the relationship between these cytokines and pyuria, bacterial growth and lower urinary tract symptoms. The primary outcome data were assessed for normality using Q-Q plots, and parametric methods of analysis used as the data were normally distributed. Data from patients and controls were pooled to compare the performance of urine cytokines in relation to pyuria, bacterial growth by enhanced sediment culture and symptoms, in patients and controls. A linear mixed-effects models analysis (SPSS) was used to analyse the data to take into account the repeated measures. Urinary IL-6 and Lactoferrin were designated as the dependent variables, and other measures entered as independent covariates in the model. The categorical data were analysed by contingency tables and the χ2 test. The analysis was undertaken with the supervision of a statistician familiar with the analysis of multilevel models in SPSS.

## Results

Between April 2011 and September 2013, 24 female patients with OAB (mean age = 63; *sd* = 11) and 22 asymptomatic control subjects (mean age 59; sd = 9) were recruited. The groups were matched for age, menopausal status and BMI. There was one drop-out from the patient group and one drop-out from the control group.

The results of the MSU cultures and dipsticks tests are reported in Tables 1 and 2. There were significant differences between the OAB patients and the normal controls in that the OAB patients were more likely to demonstrate a positive test.

**Table 1:** The results of the MSU cultures achieved over 6 two-monthly assessments

Group	Negative culture	Mixed growth	Positive culture
Normal controls	197 (89.5%)	22 (10.0%)	1 (0.5%)
OAB patients	231 (83.1%)	21 ( 7.6%)	26 (9.4%)
Chi. squared = 19.38, *df* = 2, *p* < .001

**Table 2:** The results of the dipsticks tests achieved over 6 two-monthly assessments

Group	Leucocyte negative	Leucocyte positive
Normal controls	206 (91.2%)	20 ( 8.8%)
OAB patients	172 (61.0%)	110 (39.0%)
Chi.squared = 58.35, *df* = 1, *p* < .001		

Group	Nitrite negative	Nitrite positive
Normal controls	226 (100.0%)	0 (0.0%)
OAB patients	275 ( 97.5%)	7 (2.5%)
Chi. squared = 4.008, *df* = 1, *p* = .05		

The linear mixed-effects models procedure, used to analyse the longitudinal data, explored the relationship between urinary IL-6 / Lactoferrin and urgency score, pain score, LUTS score, pyuria and microbial growth on enhanced sediment culture. In the first model the independent covariate was the group (0 or 1 / control or patient), the dependant variable IL-6 / Lactoferrin and the repeated variables were indexed on visit number.

A second model examined the pooled data from 144 OAB patient visits and 132 control visits.

The dependant variable was log IL-6 / Lactoferrin and the independent covariates were the group number (0/1; controls/patients), urgency score, pain score, LUTS score, log pyuria and log total microbial growth.

There was a significant difference in urinary IL-6 between patients and controls (Table 3) and similar differences were found with Lactoferrin Table 4.

**Table 3:** Linear mixed models analysis with IL-6 as the dependant variable

Source	Numerator df	Denominator df	F	Significance
Group	1	247	49.0	*p* < .001

Dependent variable: LogIL6

**Table 4:** Linear mixed models analysis with Lactoferrin as the dependant variable

Source	Numerator *df*	Denominator *df*	F	Significance
Group	1	255	228.5	*p* < .001

Dependent variable: LogLactoferrin

Table 5 shows the results of the mixed model repeated measures analysis applied to IL-6; it can be seen that pyuria count (log_10_ wbc µl^−1^) and however total bacterial growth (log_10_ CFU ml^−1^) proved a significant predictor of IL-6 however the symptoms scores did not.

**Table 5:** Mixed models analysis with IL-6 as the dependant variable

Dependent variable	Denominator df	F	Significance
LUTS score	205	0.w	*p* = .658
Urgency	233	1.0	*p* = .317
Pain	239	0	*p* = .935
Bacterial growth^§^	237	5.1	*p* = .024
Pyuria count^†^	258	66.2	*p* = < .001

*F is the test statistic: ratio of the variances and increase in magnitude of dependent variable demonstrated by patients compared with control subjects during the study; ^§^Bacterial growth: log cfu ml^−1^; ^†^Pyuria count: wbc ul^−1^

**Fig. 1:**
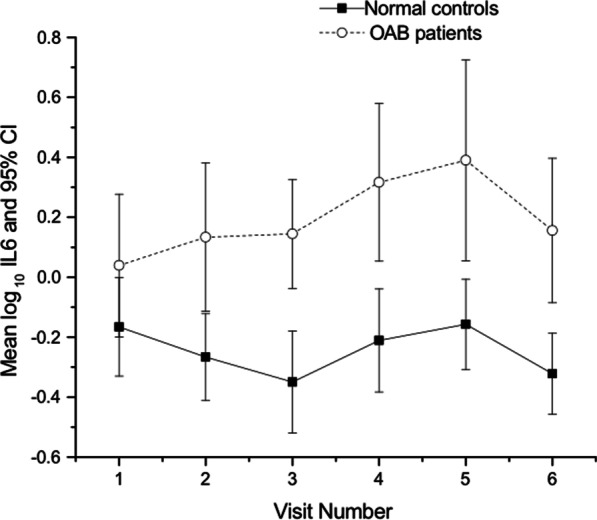
Mean Log IL-6 in patients and controls at each visit

Mean urinary IL-6 was found to be significantly higher in patients when compared with controls across each visit (Fig. 1).

The linear mixed-effects models procedure was similarly used to explore the relationship between urinary Lactoferrin and urgency score, pain score, LUTS score, pyuria and microbial growth on enhanced sediment culture. In the first model the independent covariate was the group (0 or 1 / control or patient), the dependant variable Lactoferrin, and the repeated variables were indexed on visit number. There was a significant difference in urinary Lactoferrin between patients and controls (Table 4).

A multiple mixed-effects model analysis was performed where the dependant variable was log Lactoferrin and the independent covariates were the group number (0/1), LUTS score, urgency score, pain score, log total microbial growth and log pyuria. Table 6 shows the results for each of these parameters. The predictors of Lactoferrin proved to be the LUTS symptoms, urgency and pyuria count (log_10_ wbc µl^−1^) however total bacterial growth (log_10_ CFU ml^−1^) did not.

**Table 6:** Mixed models analysis with Lactoferrin as the dependant variable

Dependent variable	Denominator df	F	Significance
LUTS score	200	9.3	*p* = .003
Urgency	232	6.6	*p* = .011
Pain	208	0.2	*p* = .622
Bacterial growth^§^	231	0.1	*p* = .793
Pyuria count^†^	240	73.9	*p* = < .001

*F is the test statistic: ratio of the variances and increase in magnitude of dependent variable demonstrated by patients compared with control subjects during the study; ^§^Bacterial growth: log cfu ml^−1^; ^†^Pyuria count: wbc ul^−1^

**Fig. 2:**
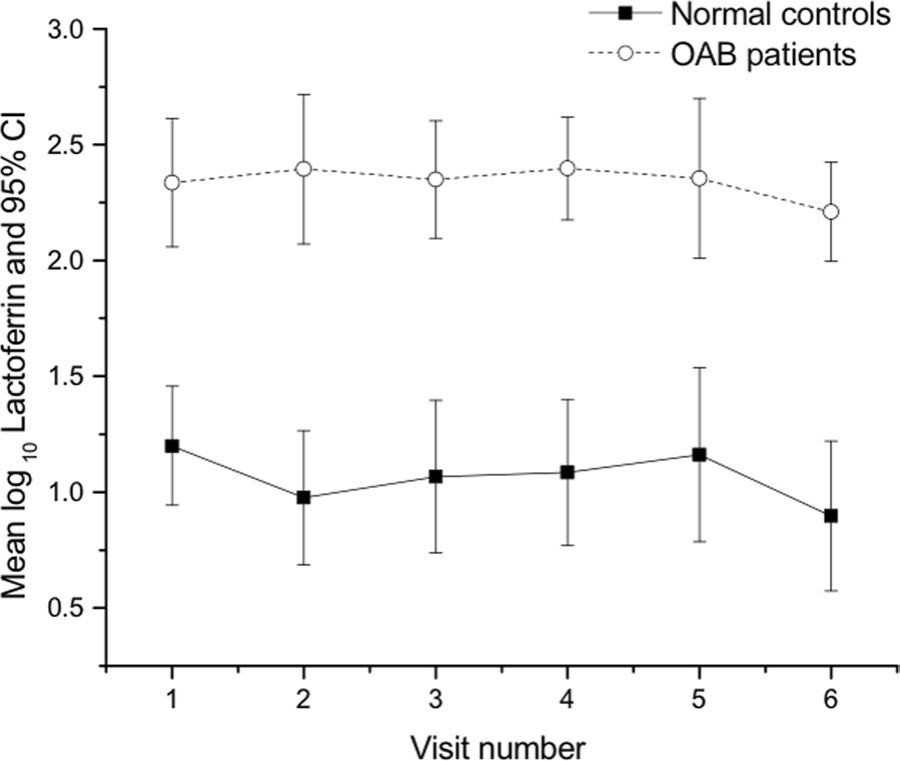
Mean Log Lactoferrin in patients and controls at each visit

Mean urinary Lactoferrin was found to be significantly higher in the patient group when compared with controls across each visit (Fig. 2).

## Conclusion

This prospective, blinded comparative study addressed the role of inflammation in the urine of patients with overactive bladder symptoms, expressed as an increase in urinary cytokines. We also measured bacterial colonisation, and markers of infection, by pathogens in this group. The data demonstrate evidence of urinary tract inflammation and this was associated with increased evidence of microbial infection in women with OAB syndrome.

These data should be interpreted with caution due to the following limitations. We followed patients over a year whilst they were attending a centre for treatment. We made no attempt to measure the effect of treatment, whether by antimuscarinics or antibiotics; the study did not have that scope. Our purpose was to test for evidence of inflammation, ascertain whether there were any correlations with markers of infection and whether the findings were consistent and persisted over a twelve month period. Treatment effects in similar patients have been studied elsewhere [18, 19] but RCT data are still outstanding. In the context of this study; intervention studies that examine treatment effects on the variables we scrutinised are still necessary. Our measures are indirect markers of inflammation, we did not study the bladder histology or cystoscopic appearances. However, these patients had undergone normal cystoscopy and renal tract ultrasound prior to their diagnosis of OAB. The measures that we used to diagnose UTI -microscopy of fresh unspun urine and sediment culture are well validated. We emphasise that the gold standard criteria for the diagnosis or exclusion of UTI using MSU culture has been shown to be incompetent at this function. Enhanced culture studies and 16 s-rRNA genomics have shown that Kass’ key assumption of a sterile normal bladder was incorrect and that his culture methods are overlooking a substantial number of infections [3, 10]. Determining causation is impossible given such a diverse microbiome with extensive overlap between patients and controls [3, 10, 20] thus we are limited to observations of association.

There is a growing body of evidence, from cross-sectional data, that bacterial infection may play a role in LUTS and OAB [6]. In this study, the patient group and controls were matched for age, BMI and menopause status and given the sample size, statistical power was maintained throughout. Inflammatory disease process in OAB patients compared to controls was clearly evident when we compared the microscopic pyuria, urinary IL-6 and Lactoferrin between the two groups. On the other hand urinary dipstick tests and MSU cultures, which are known to be insensitive [3], nevertheless differed between the two study groups. We noted a significant between groups difference in microbial abundance when we used a validated enhanced culture [10].

Urinary IL-6 association with pyuria and microbial growth did not come as a surprise; IL-6 acts as a pro-inflammatory agent attracting white cells into the tissues [21]. Increased urinary IL-6 secretion has been demonstrated in prospective studies of acute UTI [22]. Bacteria will trigger an innate immune response by parasitising uroepithelial cells. Like other mucosal surfaces, the lining of the urinary tract carries receptors capable of recognising invading microbes by binding to receptors to “Pathogen associated molecular patterns” (PAMPS). Of the various of these immune surveillance molecules the Toll-like receptor (TLR) family is the best characterised through studies of bacterial adherence and TLR4 signalling. In vivo studies, of a bacterial challenge to urothelial cells, have shown a rapid cytokine response with production of interleukin-1beta (IL-1beta), IL-6, and IL-8 (CXCL8). Type 1-fimbriated E. coli were found to activate IL-6 and IL-8 production more efficiently than the non-fimbriated[23]. IL-6 has been detected in the urine of a majority of patients with positive cultures, but it was only detected in the serum of symptomatic patients [24]. It has been argued that the cytokine response during UTI could have local and systemic components; epithelial cells are thought to be responsible for the local cytokine reaction [25]. Studies have shown that IL-6 production increases neutrophil migration and activation of IL8 [26, 27]. Uroepithelial cells also respond, not just to bacteria, but they can be stimulated by cytokines: IL-1alpha and TNFalpha induce secretion, by urothelial cells of IL-6 and IL-8 and the upregulation of mRNAs for IL-1alpha, IL-1beta, IL-6 and IL-8 [28]. There is a complex regulation of the production of IL-6 by membrane-bound and soluble receptors, which are modulated by numerous influences. Strains of *Escherichia coli* (*E.coli*) have developed strategies to evade immune response by suppressing cytokine reactions to infection [29].The studies discussed in this section have addressed acute UTI and its influence on urinary IL-6 secretion [22]; Our study takes a further step by implying a role for IL-6 in chronic disease.

Our data showed that urinary Lactoferrin was associated with pyuria and symptoms. Lactoferrin’s primary role is to sequester free iron, so it inconveniences bacteria by removing a substrate essential for bacterial growth [30]. Lactoferrin has other antimicrobial properties, a key one is by binding to the lipopolysaccharide (LPS) of the bacterial wall and then generating peroxides; these oxidise membrane molecules causing bacterial lysis [30]. Lactoferrin will stimulate phagocytes. It has been reported to interfere with proton translocation through the cell membrane, disrupting microbial chemistry and neutralising cell attachment capacity [31, 32]. Specifically, Lactoferrin interacts with membrane-LPS *E. coli*, removing LPS from the membrane [33]. The susceptibility of the bacteria to Lactoferrin is thought to be dependent on their growth phase since bacteria are more open to killing by Lactoferrin when in early phase [32]. Lactoferrin has also been shown to modulate the activity of known antibacterial agents such as lysozymes and antibiotics [34]. It is thought that the dominant mechanism of action is via its iron binding properties and interaction with LPS on Gram-negative bacteria [35].

The data from this study were collected by application of a blinded, prospective, comparative protocol. The analysis of various measures that reflect different perspectives is called consilience and it strengthens the validity of the observations because of their coherence. This work has demonstrated strong evidence of inflammation of the urothelium associated with OAB and there is good evidence that infection is playing a significant part. For many years UTI has been excluded in OAB patients by deploying tests that are insensitive. Our data present a compelling case for us to re-evaluate our understanding of OAB, detrusor instability and detrusor hyperreflexia.

## Data Availability

All data and materials with in the manuscript are the original work of the authors. The manuscript contains no third party material.

## Raw data

The datasets used and/or analysed during the current study are available from the corresponding author on reasonable request.

## References

[1] Drake MJ (2014). Do we need a new definition of the overactive bladder syndrome? ICI-RS 2013. Neurourol Urodyn.

[2] Kass EH (1957). Bacteriuria and the diagnosis of infection in the urinary tract. ArchInternMed.

[3] Sathiananthamoorthy S, Malone-Lee J, Gill K, Tymon A, Nguyen TK, Gurung S (2019). Reassessment of routine midstream culture in diagnosis of urinary tract infection. J Clin Microbiol.

[4] Kupelian AS, Horsley H, Khasriya R, Amussah RT, Badiani R, Courtney AM (2013). Discrediting microscopic pyuria and leucocyte esterase as diagnostic surrogates for infection in patients with lower urinary tract symptoms: results from a clinical and laboratory evaluation. Bju Int.

[5] Gill K, Kang R, Sathiananthamoorthy S, Khasriya R, Malone-Lee J (2018). A blinded observational cohort study of the microbiological ecology associated with pyuria and overactive bladder symptoms. Int Urogynecol J.

[6] Khasriya R, Barcella W, De Iorio M, Swamy S, Gill K, Kupelian A (2017). Lower urinary tract symptoms that predict microscopic pyuria. Int Urogynecol J.

[7] Khasriya R, Khan S, Lunawat R, Bishara S, Bignal J, Malone-Lee M (2010). The inadequacy of urinary dipstick and microscopy as surrogate markers of urinary tract infection in urological outpatients with lower urinary tract symptoms without acute frequency and dysuria. JUrol.

[8] Gill K, Horsley H, Kupelian AS, Baio G, De Iorio M, Sathiananamoorthy S (2015). Urinary ATP as an indicator of infection and inflammation of the urinary tract in patients with lower urinary tract symptoms. BMC Urol.

[9] Horsley H, Malone-Lee J, Holland D, Tuz M, Hibbert A, Kelsey M (2013). Enterococcus faecalis subverts and invades the host urothelium in patients with chronic urinary tract infection. PLoS ONE.

[10] Khasriya R, Sathiananthamoorthy S, Ismail S, Kelsey M, Wilson M, Rohn JL (2013). Spectrum of bacterial colonization associated with urothelial cells from patients with chronic lower urinary tract symptoms. J Clin Microbiol.

[11] Sathiananthamoorthy S, Malone-Lee J, Gill K, Tymon A, Nguyen TK, Gurung S (2018). Reassessment of routine midstream culture in diagnosis of urinary tract infection. J Clin Microbiol.

[12] Abrams P, Cardozo L, Fall M, Griffiths D, Rosier P, Ulmsten U (2003). The standardisation of terminology in lower urinary tract function: report from the standardisation sub-committee of the International Continence Society. Urology.

[13] Abrams P, Avery K, Gardener N, Donovan J (2006). The International Consultation on Incontinence Modular Questionnaire: www.iciq.net. J Urol.

[14] Abrams P, Andersson KE, Buccafusco JJ, Chapple C, de Groat WC, Fryer AD (2006). Muscarinic receptors: their distribution and function in body systems, and the implications for treating overactive bladder. BrJ Pharmacol.

[15] Al-Buheissi SZ, Malone-Lee J (2007). A simple well validated method for measuring urinary urgency. BJU Int.

[16] Malone-Lee JG, Al-Buheissi S (2009). Does urodynamic verification of overactive bladder determine treatment success? Results from a randomized placebo-controlled study. BJUInt.

[17] Chaliha C A-BS, Khasriya R, Khan S, Lunawat R, Bishara S, Malone-Lee J. Characterising the phenotype of the painful bladder syndrome in patients presenting with lower urinary tract symptoms. International Urogynaecological Assocation Annual Meeting; Como Italy: Springer; 2009. p. 458.

[18] Swamy S, Kupelian AS, Khasriya R, Dharmasena D, Toteva H, Dehpour T (2018). Cross-over data supporting long-term antibiotic treatment in patients with painful lower urinary tract symptoms, pyuria and negative urinalysis. Int Urogynecol J.

[19] Swamy S, Barcella W, De Iorio M, Gill K, Khasriya R, Kupelian AS (2018). Recalcitrant chronic bladder pain and recurrent cystitis but negative urinalysis: What should we do?. Int Urogynecol J.

[20] Wolfe AJ, Toh E, Shibata N, Rong R, Kenton K, Fitzgerald M (2012). Evidence of uncultivated bacteria in the adult female bladder. J Clin Microbiol.

[21] Wood MW, Breitschwerdt EB, Gookin JL (2011). Autocrine effects of interleukin-6 mediate acute-phase proinflammatory and tissue-reparative transcriptional responses of canine bladder mucosa. Infect Immun.

[22] Czaja CA, Stamm WE, Stapleton AE, Roberts PL, Hawn TR, Scholes D (2009). Prospective cohort study of microbial and inflammatory events immediately preceding Escherichia coli recurrent urinary tract infection in women. JInfectDis.

[23] Samuelsson P, Hang L, Wullt B, Irjala H, Svanborg C (2004). Toll-like receptor 4 expression and cytokine responses in the human urinary tract mucosa. InfectImmun.

[24] Hedges S, Anderson P, Lidin-Janson G, de Man P, Svanborg C (1991). Interleukin-6 response to deliberate colonization of the human urinary tract with gram-negative bacteria. Infect Immun.

[25] Agace W, Hedges S, Andersson U, Andersson J, Ceska M, Svanborg C (1993). Selective cytokine production by epithelial cells following exposure to *Escherichia coli*. InfectImmun.

[26] Sadik CD, Kim ND, Luster AD (2011). Neutrophils cascading their way to inflammation. Trends Immunol.

[27] Mihara M, Hashizume M, Yoshida H, Suzuki M, Shiina M (2012). IL-6/IL-6 receptor system and its role in physiological and pathological conditions. Clin Sci (Lond).

[28] Hedges S, Agace W, Svensson M, Sjogren AC, Ceska M, Svanborg C (1994). Uroepithelial cells are part of a mucosal cytokine network. InfectImmun.

[29] Hunstad DA, Justice SS (2010). Intracellular lifestyles and immune evasion strategies of uropathogenic Escherichia coli. AnnuRevMicrobiol.

[30] Farnaud S, Evans RW (2003). Lactoferrin–a multifunctional protein with antimicrobial properties. Mol Immunol.

[31] Andrés MT, Fierro JF (2010). Antimicrobial mechanism of action of transferrins: selective inhibition of H+-ATPase. Antimicrob Agents Chemother.

[32] Bortner CA, Arnold RR, Miller RD (1989). Bactericidal effect of lactoferrin on Legionella pneumophila: effect of the physiological state of the organism. Can J Microbiol.

[33] Appelmelk BJ, An YQ, Geerts M, Thijs BG, de Boer HA, MacLaren DM (1994). Lactoferrin is a lipid A-binding protein. Infect Immun.

[34] Ellison RT, Giehl TJ (1991). Killing of gram-negative bacteria by lactoferrin and lysozyme. J Clin Investig.

[35] Odell EW, Sarra R, Foxworthy M, Chapple DS, Evans RW (1996). Antibacterial activity of peptides homologous to a loop region in human lactoferrin. FEBS Lett.

